# Comparing the latent state–trait structure of the PANSS in cariprazine-medicated and placebo-controlled patients with acute schizophrenia

**DOI:** 10.1007/s00406-024-01790-3

**Published:** 2024-03-29

**Authors:** Jana S. Krückl, Károly Acsai, Zsófia B. Dombi, Julian Moeller, Roselind Lieb, Undine E. Lang, Ágota Barabássy, Christian G. Huber

**Affiliations:** 1 Psychiatric University Clinic (UPK Basel), University of Basel, Wilhelm Klein – Strasse 27, 4002 Basel, Switzerland; 2 Department of Psychology, Division of Clinical Psychology and Epidemiology, University of Basel, Basel, Switzerland; 3 Global Medical Division, Gedeon Richter Plc, Budapest, Hungary

**Keywords:** Latent state–trait model, Schizophrenia, PANSS, Five-factor model, Cariprazine

## Abstract

After over a hundred years of research, the question whether the symptoms of schizophrenia are rather trait-like (being a relatively stable quality of individuals) or state-like (being substance to change) is still unanswered. To assess the trait and the state component in patients with acute schizophrenia, one group receiving antipsychotic treatment, the other not. Data from four phase II/III, 6-week, randomized, double-blind, placebo-controlled trials of similar design that included patients with acute exacerbation of schizophrenia were pooled. In every trial, one treatment group received a third-generation antipsychotic, cariprazine, and the other group placebo. To assess symptoms of schizophrenia, the Positive and Negative Symptom Scale (PANSS) was applied. Further analyses were conducted using the five subscales as proposed by Wallwork and colleagues. A latent state–trait (LST) model was developed to estimate the trait and state components of the total variance of the observed scores. All symptom dimensions behaved more in a trait-like manner. The proportions of all sources of variability changed over the course of the observational period, with a bent around weeks 3 and 4. Visually inspected, no major differences were found between the two treatment groups regarding the LST structure of symptom dimensions. This high proportion of inter-individual stability may represent an inherent part of symptomatology that behaves independently from treatment status.

**Supplementary Information:**

The online version contains supplementary material available at 10.1007/s00406-024-01790-3.

## Introduction

Many researchers have previously examined the stability of schizophrenia symptoms over the time [1–5]. One approach to investigate how these symptoms change is to estimate the trait versus state aspects of different symptoms [6–12]. For instance, Chiappelli et al. [6] reported that depressive symptoms in schizophrenia patients have state as well as trait aspects in patients with schizophrenia. Maat et al. [7] found that emotional processing is also both state dependent (i.e., present during psychosis and significantly associated with symptom severity) and trait dependent (i.e., associated with the disorder across different stages). Although these studies provide evidence concerning the stability of different schizophrenia symptoms, their results are limited to one single symptom dimension in each study. Moreover, they focused on whether schizophrenia symptoms generally have a trait and/or a state component, but not how large these components are. However, both—including several symptom dimensions and estimating the size of trait versus state components—may offer valuable clues about the most effective treatment of patients with schizophrenia, e.g., which symptoms are rather variable and thus may potentially better influenceable by treatment? How large is the potential for change? On the other side, which symptoms are rather stable and are, therefore, less likely to change?

Two studies have tried to answer these questions using a latent state–trait (LST) model as introduced by Steyer et al. [13–15]. However, both studies restricted their sample to clinical high-risk state and/or first-episode psychosis individuals (and not patients with schizophrenia). Hochstrasser et al. [16] analyzed the longitudinal LST structure of the different dimensions of psychosis symptoms in clinical high-risk state and first-episode psychosis individuals over a 1-year timespan. Authors examined if the different symptom clusters (positive, negative, affectivity, resistance, activation, and excitement) according to the Brief Psychiatric Rating Scale (BPRS) differ in their trait and state characters and found that negative symptoms had the highest trait component, although positive symptoms and affectivity also exhibited high trait components [16]. The researchers concluded that an increasing proportion of psychosis symptoms persists over time [16]. Rössler et al. [17], on the other hand, applied the LST theory to sub-clinical psychosis symptoms over a 30-year time span. According to their findings, sub-clinical psychosis is predominantly influenced by stable traits at the age of 30, while before, at the age of 20, and after, at the age of 50, occasion-specific states were found to be more influential [17]. In the present study, we extend previous research by investigating a sample of patients with a pre-existing diagnosis of schizophrenia.

It is important to note that symptom variability may have several underlying causes from the natural, individual course of the disorder to independent environmental influences, e.g., antipsychotic medication [18, 19]. Nonetheless, treatment choices and outcomes can also differ on an individual level due to responsiveness to treatment and symptom combinations present as mentioned above [20, 21]. As such, it is well known that positive symptoms often respond better to antipsychotic treatment than negative or cognitive symptoms do [22, 23], while hostile or aggressive symptoms are also well addressed by antipsychotic treatment [24]. Therefore, in the present study, we compare acute schizophrenia patients with antipsychotic treatment (receiving cariprazine) to those without antipsychotic treatment (receiving placebo) in terms of the LST structure of their different symptom domains.

## Methods

### Compound and study design

Data from four phase II/III, multicenter, short-term, 6-week, randomized, double-blind, placebo-controlled cariprazine trials of similar design that included patients with acute exacerbation of schizophrenia were pooled (Clinical Trial Registration Numbers: NCT00404573, NCT00694707, NCT01104779, NCT01104779) [25–28]. Each trial started with a 1-week wash-out period which was followed by a 6-week double-blind treatment period and a 2–4-week safety follow-up. In all four trials, the primary efficacy point was changed from the baseline to week 6 in the Positive and Negative Symptom Scale (PANSS) total score [25–28]. Overall doses of cariprazine 1.5–12.0 mg were examined in comparison with placebo and two active comparators (risperidone and aripiprazole) in two studies [26, 27].

The authors assert that all procedures contributing to this work comply with the ethical standards of the relevant national and institutional committees on human experimentation and with the Helsinki Declaration of 1975, as revised in 2008. All procedures involving human subjects/patients were approved by the relevant local and/or national review board (see original articles [25–28]).

### Patients

Patients between ages 18–60 with a diagnosis of schizophrenia according to the Diagnostic and Statistical Manual 4th edition [29] who were experiencing an acute episode for less than 2 weeks were included in the studies [25–28]. In addition, participants had to have a score between 80 and 120 on the PANSS, including a score of at least 4 on more than two of the PANSS positive symptom items as well as a minimum score of 4 on the Clinical Global Impression-Severity scale. Patients were hospitalized at screening and remained in this in-patient treatment setting for at least for 3 weeks. Patients were excluded if they were suffering from another psychotic disorder or substance abuse within 3 months of the study, if it was their first episode of psychosis, or if they were treatment resistant or exhibited risk for suicide. Concomitant medications except for drugs alleviating agitation, irritability, hostility, and restlessness (lorazepam, oxazepam or diazepam), managing insomnia (eszopiclone, zolpidem, zolpidem extended release, chloral hydrate, or zaleplon) or treating extrapyramidal symptoms (diphenhydramine, benztropine or equivalent, or propranolol) were prohibited. Patients that were randomized to risperidone or aripiprazole were excluded from the current analyses. The current post hoc analysis was based on a subset of patients who completed the study without any missing data. We built two treatment groups based on this subset: a cariprazine group (CAR; treated with any dose of cariprazine) and a placebo group (PBO).

### Assessment

The Positive and Negative Syndrome Scale (PANSS) is a widely used scale to assess symptoms of schizophrenia. Although there is a continuous debate regarding the factor structure of the PANSS [30–32], one of the most accepted constructs is the five-factor model by Wallwork et al. [32]. According to this concept, schizophrenia spectrum symptomatology is composed of five symptom dimensions: positive symptoms, negative symptoms, cognitive symptoms, excitement–hostility symptoms, and anxiety–depression symptoms [32]. To derive a consensus model, Wallwork et al. [32] have attributed those items (20 out of the 30) to the 5-symptom dimensions which have been found to load on this factor in most published factor analyses.

### Statistical analyses

An LST model was developed to estimate the trait and state components of the total variance of the observed scores. LST models are one of the most commonly applied modeling approaches for understanding the observed variability in longitudinal clinical studies, within the general framework of structural equation modeling (SEM). In SEM, just like in simple regression, the relationships between dependent and independent variables are studied, by estimating the explanatory power of the independent variable(s) on the dependent variable(s). In other words, we study the degree to which the variabilities of the dependent variables can be explained by the independent variables. In addition to terminology used in general regression analyses, variables in the SEM framework can also be classified into the categories of latent and observed variables. Latent variables cannot be measured directly (e.g., intelligence, or state and trait variables in an LST model for a psychiatric disease). Instead, they can be measured indirectly, through the observed (indicator) variables (e.g., math scores or reading comprehension in students, or specific psychometric scores in schizophrenia patients). Thus, SEM can be viewed as a generalization of the simple regression analysis, with handling a rather difficult network of observed and latent variables, where one of the possible purposes is to test different hypotheses about the relationships between the variables. Despite the analogy, the LST state and trait variables may remain less intuitive compared to the general intelligence. Continuing with the analogy, intelligence can be regarded as a stable component, which basically determines the student’s achievements in different circumstances. The actual mental achievements, however, can obviously be affected by situational (state) factors, just like in cases of psychiatric diseases. The purpose of the LST analysis is to estimate the relative contributions of the trait and state factors to the observed variables, resulting presumably in helpful conclusions regarding the treatment of the disease. For readers interested in the thorough discussion of commonly applied models by SEM, including LST models, we refer to excellent textbooks in the “References” section. In the model, trait variability represents the inter-individual differences within the population, while the state component reflects the effects that are due to the situation and the interactions between the person and the situation. For model building, the general strategy described by Newsom [33] was followed.

Altogether, seven latent state variables were defined corresponding to the number of visits during the study (baseline, week 1, week 2, week 3, week 4, week 5, and week 6), while a single latent variable represented the trait factor (see Supplementary Fig. 1). Each of these latent state variables were defined by the five Wallwork factors as observed indicator variables.

The loadings for the second-order trait factor from the occasion-specific state factors were set to 1 resulting in equal contributions. Effects coding identification approach was used in the estimation of the loading parameters. This approach estimates the parameters based on a weighted function of all loadings and observed variances of the indicators.

The model fit including only latent state and trait variables was poor. Therefore, latent method specific factors with the indicator variables were defined, to account for the possible differences between the covariance patterns within and between the scores across the visits. This resulted in substantial improvement in the model fit, but still remaining under the acceptable level. Thus, further correlations were allowed to be estimated based on the modification indices, which indicated considerable correlations mainly between the observed variables adjacent in time. After refitting the model, the fit indices reached acceptable levels with a Comparative Fit Index Goodness-of-fit index (CFI) = 0.95 (0.84 before refit; a value >0.90 reflects an acceptable fit) and a Root-Mean-Square Error of Approximation (RMSEA) = 0.065 (0.085 before refit; values closer to 0 represent a good fit, should be <0.08)).

Consistency (CO) and occasion-specific (OS) coefficients were calculated according to Newsom [33], using averaged parameters as appropriate and expressed as percentages of the total variability. The CO coefficient quantifies the degree of inter-individual stability across situations; the larger it is, the more inter-individually similar scores vary across specific situations or time points [33, 34]. In contrast, the OS coefficient indicates the degree to which individual differences on the observed variables are determined by the situation or by person–situation interactions [34]. Thus, the larger the OS coefficient, the stronger the situation-specific influences on the observed scores [34].

The model was calculated for both treatment groups, the CAR group (receiving cariprazine) and the PBO group (receiving placebo), individually. With regard to the exploratory and descriptive approach in the present study, we refrained from inferential statistical comparisons between the two models. The model estimations were done using the R package, Lavaan [35].

## Results

### Baseline characteristics

The total number of patients included in the analysis was 1138 (CAR: 807; PBO: 331). The number of parameters to be estimated was 222, thus meeting the generally accepted requirement that ratio of *N*/parameters should be greater than or equal to 5.

In the CAR group, there were 570 men (70.6%), and the mean age was 38.1 years (std. deviation *σ* = 10.5). On average, patients were diagnosed with schizophrenia 12.5 years beforehand (*σ* = 10.5, min = 0.7, max = 49.2). In the PBO group, 236 patients were male (71.3%). The mean age was 38.4 years (*σ* = 10.9). The mean duration of schizophrenia was 13.1 years (*σ* = 10.4, min = 0.8, max = 46.0). For more clinical information, see Table 1.

**Table 1:** Baseline characteristics

	Total group	Medicated group (CAR)	Unmedicated group (PBO)	Group comparisons
*N*	1138	807	331	
Gender (male)	806 (70.8%)	570 (70.6%)	236 (71.3%)	*χ*^2^(1) = 0.05, *p* = .82
Age	38.1 (10.6)	38.1 (10.5)	38.4 (10.9)	*t* (1136) = −0.42, *p* = .68
Duration of schizophrenia (in years)	12.7 (10.0)	12.5 (9.8)	13.1 (10.4)	*t* (1136) = −0.90, *p* = .37
Schizophrenia type				
295.10 Disorganized type	32 (2.8%)	21 (2.6%)	11 (3.3%)	*χ*^2^(3) = 1.53, *p* = .67
295.20 Catatonic type	1 (0.1%)	1 (0.1%)	0 (0.0%)	
295.30 Paranoid type	1010 (88.8%)	721 (89.3%)	289 (87.3%)	
295.90 Undifferentiated type	95 (8.3%)	64 (7.9%)	31 (9.3%)	
PANSS scores				
Positive symptoms (POS)	15.4 (2.9)	15.4 (2.9)	15.5 (2.9)	ES = 0.01, *p* = .82
Negative symptoms (NEG)	19.4 (4.2)	19.3 (4.2)	19.6 (4.3)	ES = 0.11, *p* = .11
Disorganized thoughts (DIS)	10.8 (2.3)	10.8 (2.2)	10.6 (2.5)	ES = 0.12, *p* = .08
Uncontrolled hostility/excitement (EX)	10.3 (3.2)	10.4 (3.1)	10.0 (3.2)	ES = 0.13, *p* = .04
Anxiety/depression (DE)	8.2 (2.6)	8.2 (2.5)	8.3 (2.7)	ES = 0.04, *p* = .54
Symptom severity (PANSS total score)				
Moderately ill (score between 59 and 75)	2 (0.2%)	1 (0.1%)	1 (0.3%)	*χ*^2^(3) = 0.54, *p* = .91
Markedly ill (score between 76 and 95)	587 (51.6%)	415 (51.4%)	172 (52.0%)	
Severely ill (score between 96 and 116)	530 (46.6%)	378 (46.8%)	152 (45.9%)	
Very severely ill (score between 117 and 210)	19 (1.7%)	13 (1.6%)	6 (1.8%)	

Note: Data is indicated as n (%) or mean (SD)

### Latent state–trait model

Among the model fit indices, Chi square value was 3917 with 1066 degrees of freedom. Thus, their ratio was lower than 5, which is generally accepted as good fit. Additional fit indices were also in acceptable ranges: the CFI = 0.95, RMSEA = 0.065.

In Table 2, the estimated model coefficients based on averaged parameters across weeks and subscales are shown. In both treatment groups and across all factor scores, the proportion of stability (CO%) is higher than occasion-specific influences (OS%). While the former ranges between 44.2 and 52.2% in the CAR group and between 45.0 and 53.8% in the PBO group, the values for the latter lie between 2.3 and 9.6% in the CAR group and 1.7% and 8.2% in the PBO group. Method variance appears to explain around a third of the total variance independent of the subscales or groups.

**Table 2:** LST model coefficients averaged across weeks and Wallwork factor scores

	CO %	OS %	Method %	Error %
CAR				
POS	47.4	5.1	33.6	13.9
NEG	50.7	8.1	29.9	11.3
DIS	52.2	9.6	30.6	7.6
EX	46.7	4.5	29.2	19.6
DE	44.2	2.3	36.1	17.4
PBO				
POS	48.6	4.3	36.0	11.1
NEG	53.3	7.9	29.2	9.6
DIS	53.8	8.2	30.1	7.9
EX	47.5	3.5	33.6	15.4
DE	45.0	1.7	38.1	15.2

*CO* consistency coefficient, *OS* occasion-specific coefficient, *POS* positive factor, *NEG* negative factor, *DIS* disorganized/concrete factor, *EX* excited factor, *DE* depressed factor

**Fig. 1:**
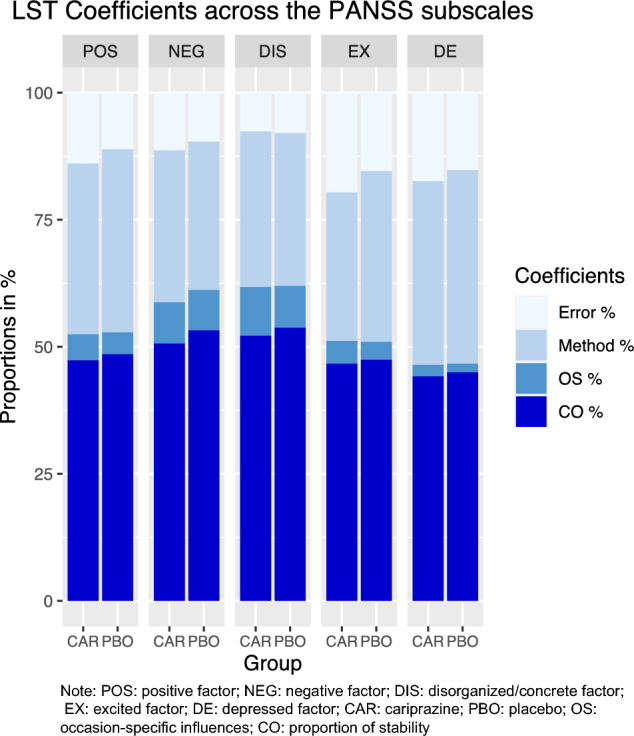
LST model coefficients averaged across weeks and Wallwork factor scores

In Fig. 1, these findings are visually displayed. The proportion of inter-individual stability is higher than occasion-specific influences across all subscales and in both treatment groups. Besides small differences, the proportions appear to be surprisingly similar comparing the CAR and the PBO group.

**Fig. 2:**
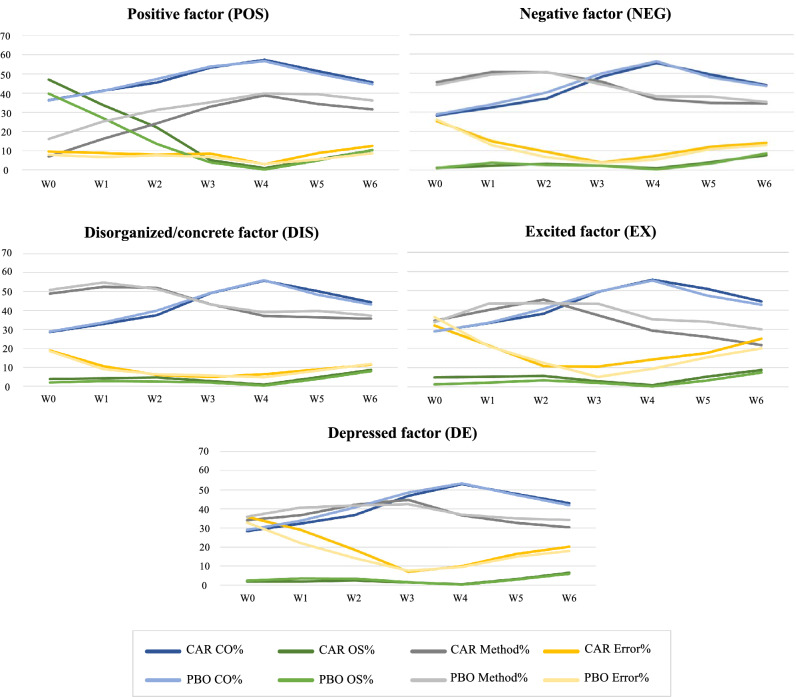
Calculated LST model coefficients by treatment group and weeks score

Figure 2 shows the changes of the CO coefficients, OS coefficients, method variance and error variance across the weeks for each subscale individually. Focusing on the weekly changes in the CO coefficients, a clear trend emerges; all subscales show an increase in the CO coefficient proportions from baseline until the third week, before they decrease again.

The variance of situation-specific influences appears to be substantially smaller than the proportion of inter-individual stability, similarly in both treatment groups. Overall, the influences of the situation and/or the interaction between person and situation are rather small.

The behavior of the OS coefficients across the seven study visits appears to be also similar in both treatment groups. During the first weeks, it decreases until week 3, then it increases again. Generally, there seems to happen a change around weeks 3/4. The proportion of unexplained variance (error variance) changes strongly across the observation period. At some time points, the ratio of unexplained variance is as high as over 30%; at others’, it is below 10%. Similar to the OS coefficients, the error variance decreases at the beginning until week 3 or 4 and increases again thereafter. The method variance shows the same development as the OS coefficient and the error variance, albeit in the opposite direction.

More detailed parameters estimations are found in the Supplementary Table 3.

## Discussion

To the knowledge of the authors, this is the first analysis that examines the LST structure of the five-factor model [32] of schizophrenia symptoms comparing patients receiving antipsychotic treatment to those without antipsychotic treatment. All symptom dimensions behaved more in a trait- rather than a state-like manner across the whole observation period of 6 weeks. Occasion-specific fluctuations appeared to have a negligible effect on how schizophrenia symptoms change over time. The proportions of all sources of variability changed over the course of the observational period, with a bent around weeks 3 and 4. Visually inspected, no major differences were found between the two treatment groups regarding the LST structure across all symptom dimensions. Among the small differences that were found, the group receiving placebo showed a slightly higher proportion of inter-individual stability. Patients receiving cariprazine, however, tended to have a larger proportion of error variance.

Previous studies investigating the stability of schizophrenia symptom dimensions have addressed trait versus state aspects of single symptoms, however, without quantifying the proportion of these aspects [6–12]. Therefore, the aim of the present study was to quantify these proportions using an LST model as introduced by Steyer et al. [13, 14].

Previously, two studies applied the LST model in the context of psychotic symptoms in clinical high-risk state and first-episode psychosis samples [16, 17]. Hochstrasser et al. showed that the proportion of variability explained by stable traits differed between symptom dimensions (importantly, “trait” referring to inter-individual (and not absolute) stability). While positive and negative symptoms exhibited high trait proportions (72.2% resp. 81.1%), only 13.2% of the variance of excitement component was explained by trait differences [16]. Since, in the present study, all symptom dimensions showed similar proportions of trait variability, our results contradict the abovementioned findings. Nonetheless, it is important to note that the observation period was different in the Hochstrasser and in the present study (1 year versus 6 weeks) which could provide an explanation to the difference. Indeed, symptoms like excitation can fluctuate in the long run, while being stable during acute episodes which last a few weeks to months [36, 37]. Our findings are, therefore, not generalizable to the long-term symptom course. Negative symptoms, on the other hand, might become more prominent after acute psychosis as reported by several other research groups [4, 38, 39]. Yet, Hochstrasser and colleagues have not analyzed the proportion of explained variance by latent states, measurement errors or method effects. Systematic changes in symptom scores may have, therefore, mistakenly attributed to trait differences which hinders in-depth comparisons between the two studies in this regard.

In contrast to Hochstrasser and colleagues, Rössler et al. [17] also reported the change of state aspects. Rössler and colleagues investigated the sub-clinical psychotic symptoms in a clinical high-risk state and first-episode psychosis population. They found that these symptoms are predominantly influenced by stable traits at the age of 30, while before and after occasion-specific states were influential [17]. Contrary to these findings, we found that situational fluctuations and/or person–situation interactions between study visits have a relatively negligible effect on how schizophrenic symptoms change over time. Averaged across the observation period, no proportion of state variability exceeded 10% in our study, while Rössler et al. [17] reported proportions up to 61.2%. This may be due to similar reasons as mentioned above; given the short observation period of our study, some symptomatic fluctuations may only become visible in the long run. Another reason for such difference may be explained by the different samples. While Rössler et al. investigated a highly diverse sample including healthy controls, clinical high-risk state individuals as well as patients with first-episode psychosis, this study included a more homogenous sample of acute schizophrenia patients. Therefore, the heterogeneous population might have led to a high diversity in terms of symptomatic change and the more homogenous and highly standardized treatment setting resulted in less fluctuation between visits. Indeed, previous research also showed that up to 90% of the sub-clinical psychotic symptoms are transitory and disappear over time [40].

It is important to note, however, that there are also similarities between the three LST studies. For instance, a large proportion of variance was explained by latent traits. Thus, the symptomatic change appears to be inter-individually rather similar than different comparing patients to each other. This overlap between the studies is surprising, especially regarding the major differences concerning sample structure, treatment settings, and duration of the observation period. This underlying trait aspect may be interpreted as a “common ground” of the assessed symptom dimensions, a structure that is shared by all symptom dimensions. As Hochstrasser et al. [16] describes it: the disorder always is “part of a ‘whole’ of mutually implicative, interpenetrating experiences, feelings, beliefs, expressions, and actions, influenced by the biographical background of the individual.” (p. 6). Previous studies also support this idea by reporting a strong inter-connectedness between schizophrenia symptoms [38, 41–43]. Moreover, although there is a high heterogeneity between schizophrenia patients, the long-term course of the symptoms appears to be rather stable at an individual level (i.e., symptoms having a high inter-individual stability). Cannon [44] introduced the idea of schizophrenia being a trait, referring to a long-term vulnerability to psychosis which, in turn, can be seen as a state. A trait aspect that is shared by all symptom dimensions may, therefore, represent the underlying structure of chronic schizophrenia that may become visible in other psychological and (neuro)biological markers [16]. However, to test this hypothesis, more research is necessary, especially including patients with other psychotic disorders (like schizoaffective disorder or delusional disorder).

This argumentation is somewhat supported by another finding of our study: visually inspected, there were no major differences between the two treatment groups (cariprazine versus placebo) regarding their LST structure. This finding might seem counterintuitive. However, the similarity in their LST structure might display something inherent to the disorder as discussed above. However, there are also other possible explanations for this observation. For example, “treatment” cannot be narrowed down to psychopharmacological medication alone. All patients have been treated in an in-patient psychiatric setting with all its numerous therapeutic effects. This may have led to a fairly similar improvement of symptom scores and similar proportions of traits and states, independently whether patients received antipsychotic treatment or not. It is, therefore, difficult to come to a conclusion regarding this finding.

Further, we found some small differences between the two treatment groups. The group receiving placebo showed a slightly higher proportion of inter-individual stability. Patients receiving the antipsychotic, however, tended to have a larger proportion of error variance. Both findings may be due to the fact that patients differ in their response to their received treatment; patients receiving the placebo tended to exhibit a weaker symptom reduction (their symptoms remain rather inter-individually stable across time), while patients receiving the antipsychotic respond stronger but possibly inter-individually more different to the medication (leading to a higher proportion of unexplained variance). However, the group differences were small, and it remains unclear whether these differences would reach clinical significance.

Finally, the trait components in our study exhibited the lowest values at the beginning, then rising to the maximum value before decreasing again. Changes of the state components behaved in an opposite pattern (U-shape). This bent at weeks 3–4 could be ascribed to the study setting as patients who all have been treated in an in-patient setting at first were allowed to leave the hospital after 3 or 4 weeks, leading to higher differing treatment settings and a, therefore, stronger varying symptom development. However, this remains as the only one potential explanation for this finding, which should be further explored in future research.

One of the main limitations of the analysis is that the data are from four phase II/III clinical trials which all had a rigorous inclusion criterion such as having a score between 80 and 120 on the PANSS, including a score of at least 4 on more than 2 of the PANSS positive symptom items. Therefore, the total variance in PANSS scores is somewhat restricted compared to what could be seen in a real-world clinical practice. Second, it cannot be ruled out that certain psychiatric comorbidities (like personality or affective disorders) or the effects of concomitant medications (like drugs alleviating agitation, or hostility) may have systematically influenced the changes in symptom scores. In addition, the timeframe of the studies was short, which again limits the unfolding of whether a symptom dimension is rather a trait or a state. This makes any conclusion about long-term variability of symptoms impossible, as state aspects of some symptoms might have only become apparent after the 6 weeks long period. Future research should, therefore, include a longer observation period. Finally, those who have dropped out from the trials were excluded from the analysis, which might bias the results in the direction of underestimating the trait components.

In summary, the present analysis sheds light on the notion that symptoms of schizophrenia appear to be rather inter-individually stable than state-like in patients suffering from an acute phase of schizophrenia. This observation was found in both—patients who received antipsychotic medication and patients who did not.

### Supplementary Information

Below is the link to the electronic supplementary material.

Supplementary file1 (PDF 133 KB)

## Data Availability

Not applicable to this article as no new data were created or analyzed in this study.
