# Supplementary material for: Comparing the latent state–trait structure of the PANSS in cariprazine-medicated and placebo-controlled patients with acute schizophrenia
Source: Eur Arch Psychiatry Clin Neurosci. 2024 Mar 29;274(6):1333–41. doi: 10.1007/s00406-024-01790-3 (PMC11362189; doi:10.1007/s00406-024-01790-3)
Supplement: Supplementary file 1 — Supplementary file1 (PDF 133 KB) [file 406_2024_1790_MOESM1_ESM.pdf]

SUPPLEMENTAL MATERIAL

Supplementary figure 1. Latent state-trait model.

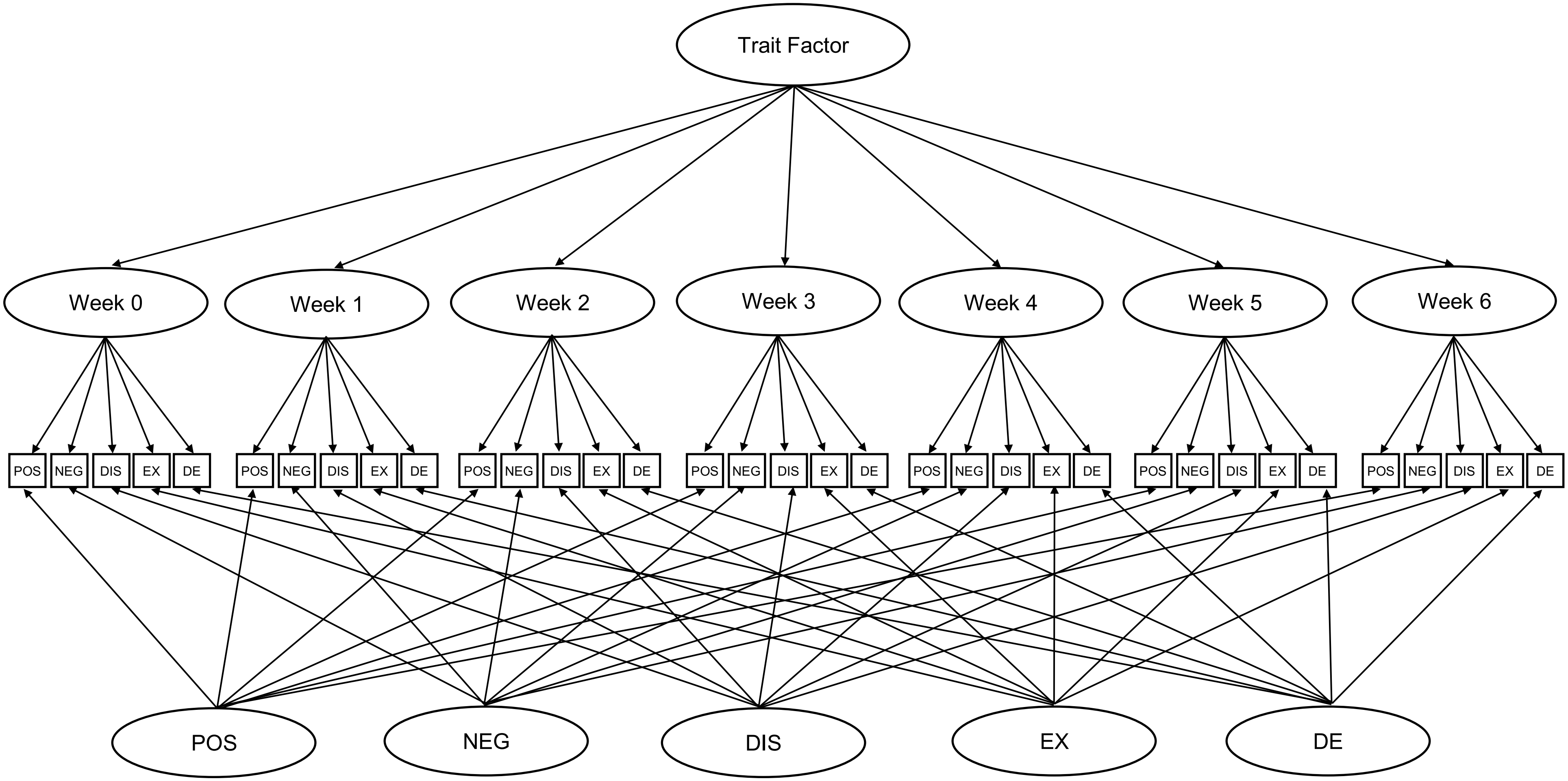

Note: The model consists of one single latent variable representing the trait factor, seven latent state variables corresponding to the number of visits during the study (baseline, week 1, week 2, week 3, week 4, week 5 and week 6), and five latent state variables defining the five Wallwork factors (POS, NEG; DIS, EX, DE) as observed indicator variables.

Supplementary table 3. LST model coefficients by treatment and score

Treatment group	Wallwork factor	Study vist	CO %	OS %	Method %	Error %
**CAR**	**POS**	Week 0	41.8	9.4	20.4	28.4
		Week 1	45.4	5.9	27.8	20.9
		Week 2	50.4	3.4	32.6	13.6
		Week 3	54.9	0.5	37.0	7.6
		Week 4	49.8	3.5	42.2	4.5
		Week 5	44.6	7.1	38.3	10.0
		Week 6	41.3	9.1	35.7	13.9
	**NEG**	Week 0	44.1	13.8	22.7	19.4
		Week 1	48.3	8.9	28.0	14.8
		Week 2	53.1	4.9	31.7	10.3
		Week 3	58.8	0.9	36.4	3.9
		Week 4	53.5	5.8	33.8	6.9
		Week 5	48.1	11.8	28.6	11.5
		Week 6	44.6	15.8	26.2	13.4
	**DIS**	Week 0	45.5	16.4	26.4	11.7
		Week 1	49.9	10.5	31.3	8.3
		Week 2	54.9	5.9	33.5	5.7
		Week 3	59.9	1.0	33.5	5.6
		Week 4	55.2	6.8	32.2	5.8
		Week 5	49.9	14.2	28.8	7.1
		Week 6	46.2	18.8	26.1	8.9
	**EX**	Week 0	40.5	6.8	19.2	33.5
		Week 1	44.1	4.6	24.3	27.0
		Week 2	49.3	2.8	30.7	17.2
		Week 3	54.4	0.5	35.6	9.5
		Week 4	49.4	3.3	35.3	12.0
		Week 5	44.6	7.0	32.3	16.1
		Week 6	41.3	9.1	25.9	23.7
	**DE**	Week 0	38.8	3.6	29.6	28.0
		Week 1	41.9	2.3	33.2	22.6
		Week 2	46.7	1.3	35.9	16.1
		Week 3	51.6	0.2	40.0	8.2
		Week 4	46.7	1.6	41.2	10.5
		Week 5	42.1	3.7	38.1	16.1
		Week 6	39.3	5.2	33.2	22.3
**PBO**	**POS**	Week 0	43.0	9.4	23.6	24.0

	Week 1	46.4	5.6	32.0	16.0
	Week 2	52.1	2.4	35.0	10.5
	Week 3	55.5	0.3	39.7	4.5
	Week 4	51.0	2.3	41.3	5.4
	Week 5	46.6	5.2	39.5	8.7
	Week 6	41.9	8.7	39.0	10.4
**NEG**	Week 0	46.0	14.6	21.9	17.5
	Week 1	50.6	9.3	28.2	11.9
	Week 2	56.5	4.1	32.1	7.3
	Week 3	60.6	0.5	34.7	4.2
	Week 4	56.6	4.6	32.5	6.3
	Week 5	51.7	10.4	27.8	10.1
	Week 6	46.5	17.0	25.7	10.8
**DIS**	Week 0	46.5	15.5	26.1	11.9
	Week 1	51.1	9.7	31.5	7.7
	Week 2	56.8	4.2	32.6	6.4
	Week 3	60.8	0.5	33.0	5.7
	Week 4	57.6	5.0	32.2	5.2
	Week 5	52.5	11.1	27.9	8.5
	Week 6	46.8	17.6	25.1	10.5
**EX**	Week 0	41.3	6.4	24.5	27.8
	Week 1	44.7	4.1	30.0	21.2
	Week 2	50.5	1.8	35.2	12.5
	Week 3	54.3	0.2	39.6	5.9
	Week 4	50.5	2.1	38.7	8.7
	Week 5	46.1	4.7	35.1	14.1
	Week 6	41.7	8.1	30.3	19.9
**DE**	Week 0	39.4	3.1	32.6	24.9
	Week 1	42.3	2.0	36.9	18.8
	Week 2	48.1	0.8	38.9	12.2
	Week 3	51.7	0.1	41.2	7.0
	Week 4	48.0	1.0	41.4	9.6
	Week 5	43.8	2.4	38.0	15.8
	Week 6	39.6	4.4	35.4	20.6

Note: standardized values, calculated based on effects coding identification. CAR: cariprazine treatment group; PBO: placebo treatment group. POS: positive symptom factor scores; NEG: negative symptom factor scores; DIS: disorganized thought factor scores; EX: uncontrolled hostility/excitement factor scores; DE: depression/anxiety factor scores. CO: Consistency coefficient; OS: Occasion specific coefficient.
